# Supplementary material for: Artificial neural network in the discrimination of lung cancer based on infrared spectroscopy
Source: PLoS One. 2022 May 12;17(5):e0268329. doi: 10.1371/journal.pone.0268329 (PMC9098097; doi:10.1371/journal.pone.0268329)
Supplement: Attachment — Submitted filename: Response to Editor and Reviewers.docx [file pone.0268329.s002.docx]

**RESPONSE TO REVIEWERS**

**Manuscript No. PONE-D-21-23770R1**

***“*Artificial neural network in the discrimination of lung cancer based on infrared spectroscopy*”***

We would like to thank the Editor and the three reviewers for the careful reading of our manuscript and for the constructive, positive, as well as critical comments.

Below, please find our responses to all the comments indicating the specific changes made to the manuscript. Our *responses* are highlighted in *italic* letters and the revisions made in the manuscript are in blue font.

**Editorial requests:**

**Comment:**

Thank you for submitting your manuscript to PLOS ONE. After careful consideration, we feel that it has merit but does not fully meet PLOS ONE’s publication criteria as it currently stands. Therefore, we invite you to submit a revised version of the manuscript that addresses the points raised during the review process.

The main points of contention from the previous reviews are the missing citation from Abbas. If the authors still disagree with the citation, I highly recommend justification with respect to the new comments by Reviewer 1. In addition, Reviewer 3 brings up some new points regarding data processing and consistency that are important to address.

**Our answer:**

*We thank the editor for the above comment. The manuscript has been edited according to the reviewers’ comments. Moreover, we have included brief responses to the reviewers’ questions.*

**Reviewer Comments:**

**Reviewer #1:**

$-$I am satisfied with the answers to comments 1 and 2. However, as regard to the answer for comment 3 I have some concern. My main concern is that, some recent previous works on lung cancer diagnosis are missing in this manuscript. The authors responded to comment 3 that “Abbas et. al. 2018 should not be included into the references because they utilized pleural fluid samples to discriminate malignant pleural mesothelioma from lung cancer and benign pleural effusion. We believe that it cannot be compared to our results because of the difference in objective and specimen used”. However, in the introduction part of the manuscript there are references related to thyroid cancer, ovarian cancer, breast cancer, image base diagnosis, and some infrared papers on lung cancer etc. which have different objectives and specimens.

On the other hand, when you examine Abbas et al’s paper not only mesothelioma samples are discriminated from lung cancer, but in addition, lung cancer samples are discriminated from both benign samples and mesothelioma samples using unsupervised and supervised methods. (please see Fig. 3 of that paper). Therefore the recent paper of Abbas et al. J. Biomed. Optics, 2018 paper should be cited

**Our answer:**

*We thank the reviewer for the comments given above. We have read the article carefully and added the paper of Abbas et al. in our* ***discussion*** *which can be seen in* ***lines ­­812-817 of page 40****:*

“Another study by Abbas *et al*. differentiates malignant pleural mesothelioma (MPM) from lung cancer as well as benign pleural effusion (BPE) using the FTIR spectral data from pleural samples wherein hierarchal cluster analysis (HCA) and PCA was applied. Their model was 100% sensitive and specific in differentiating MPM from lung cancer, and 100% sensitive and 88% specific in differentiating MPM from BPE (n=69; BPE n=25; LC n=20; MPM n=24) [92]. To further advance these findings, more sophisticated computational tools such as NNs may be used in the discrimination of spectral data. The designed NNs of this study are 89.84% to 92.87% sensitive (RR) and 86.63% to 96.01% specific (SR). While the sensitivity and specificity of Kaznowska *et al.*’s study can reach 99% [70], it has higher variability in discriminating samples compared to the NNs. Thus, SVM or NN-aided diagnosis using infrared spectroscopy may be utilized and is a better adjunct tool in identifying truly benign or malignant tissue samples.”

**Reviewer #3:**

$-$Finding optimal NN model and optimal parameters of NN by using grid search with GA on spectroscopic data is the main idea of this manuscript which has been appropriately implemented here. As per the manuscript, data consists of 122 spectral vectors; it is not mentioned how these spectra are collected. Are these collected from 122 different patients/tissue blocks? If each spectral belongs to a separate tissue block, how did you select signal spectral vector to represent the entire benign tissue or tumor part of malignant tissue? Did you collect more spectra and then take the average? With FTIR spectral collection, spectral variance is observed depending on many factors like underlying histology class in the tissue or sample density at that location.

**Our answer:**

*We thank the reviewer for the comment above to improve the process of collection. We would like to clarify that each of the 122 spectral vectors was derived from the average of 48 scans from each of their respective tissue block. Thus, the revised* ***‘Study population and sample preparation’ section, in lines 144-145 of page 6*** *now reads:*

“FTIR spectral data (n =122; 56 malignant, 66 benign) of FFPE lung biopsies from 112 adult patients seen at MMMH-MC and USTH from 2015 to 2017 comprised the dataset.”

*Additionally,* ***lines 160-163 of page 7*** *now reads:*

“All 122 tissue sections were examined in the mid-IR region (4000 cm^-1^ to 600 cm^-1^) with an average of 48 scans added to obtain an adequate signal-to-noise ratio (Zhang, 2011; Wu, 2016, Bhosale, 2011), yielding 122 spectral data with a resolution of 4 cm^-1^. This is further supported by the software’s validation program as “acceptable”.”

$-$I agree with the other reviewers that instrument parameters or environmental factors can affect spectral profile; hence proper pre-processing like baseline correction and peak normalization (not unit normalization) is needed.

**Our answer:**

*We thank the reviewer for the valuable comment regarding normalization. We acknowledged and agreed to change the normalization process to peak normalization; hence, the PCA diagrams and the median ATR-FTIR absorbance spectra of malignant and benign lung tissue samples were changed accordingly, including the discussion of such results. However, similar to the previous revision, we no longer saw the need to change our normalization in training our models since the normalization process is similarly preferable for neural networks.*

***Thus, lines 194-203 of pages 8-9 concerning Pre-processing of spectral data now read:***

“Normalization and baseline correction comprised the pre-processing of the spectral data set, $X_{SD}$. All spectral data were normalized using peak normalization before peak analysis and spectrum visualization. The normalization was performed to eliminate bias from y-value discrepancies among the IR samples due to environmental noise and instrument parameters. The peak normalization equation is as follows

$X_{SD}^{(i)} = \frac{X_{SD}^{(i)}-min(X_{SD}^{(i)})}{max(X_{SD}^{(i)})} \forall sample i$	(1)

where the $min(X_{SD}^{(i)})$ and the $\max(X_{SD}^{(i)})$ terms refer to the minimum and the maximum absorbance of the $i^{th}$ spectral data, respectively. Note that the normalization was done per spectral data element/vector $X_{SD}^{(i)}$; hence the spectrum from other spectral vector does not influence the results of normalizing another spectral vector.”

***Fig 5 was changed as follow:***

**Fig 5.** Median ATR-FTIR absorbance spectra of malignant (n = 53) and benign (n = 65) lung tissue samples. The figure shows the median FTIR spectrum of malignant and benign lung tissue samples and their corresponding peaks identified via visual analysis.

*Due to the modification in the normalization process, the mean absorbances and the p-values of each peak position for both malignant and benign samples were changed accordingly.* ***Thus, Table 3 was changed as follow:***

**Table 3.** Computation of the spectrum variables (peak positions and normalized absorbances) of malignant and benign lung samples in the fingerprint IR region (1800 cm^-1^ to 850 cm^-1^).

| **Malignant Samples**  **(n = 53)** | | **Benign Samples**  **(n = 65)** | | **Functional Group** | **Vibrational Mode** | **Molecular Source** [43,51–55] | ***p*-value*** |
| --- | --- | --- | --- | --- | --- | --- | --- |
| **Peak Position** | **Mean abs ± SD** | **Peak Position** | **Mean abs ± SD** |  |  |  |  |
| 1636 | 0.9605 ± 0.0856 | 1638 | 0.9885± 0.0139 | O=C–N–H | *v*(CO), *v*(CN) | Amide I, protein | 0.6991 |
| **1537** | **0.7884 ± 0.0898** | **1535** | **0.8585 ± 0.0491** | **O=C–N–H** | **γ(N–H), ν(C–C), ν(C–N)** | **Amide II, protein** | **<< 0.05** |
| 1452 | 0.3659 ± 0.0687 | 1452 | 0.3714± 0.0510 | –(CH2)_n_, –(CH3)_n_– | δ_as_(CH3), δ_as_(CH2), δ_s_(CH3) | Lipids | 0.9851 |
| 1401 | 0.3244 ± 0.0694 | 1397 | 0.3480 ± 0.0542 | –(CH2)_n_– | δ_s_(CH3) | Lipids | 0.0590 |
| **1314** | **0.1334 ± 0.0582** | **1312** | **0.1711 ± 0.0591** | **-** | **-** | **-** | **<< 0.05** |
| 1236 | 0.165 ± 0.0766 | 1236 | 0.1921 ± 0.0775 | RO–PO2^−^–OR | *ν*_as_(PO2^−^) | DNA, RNA, phospholipids | 0.1138 |
| 1160 | 0.0467 ± 0.0343 | 1160 | 0.0506 ± 0.0475 | C–O–H | ν(CO), γ(COH) | Carbohydrates | 0.4302 |
| **1051** | **0.3131 ± 0.1274** | **1030** | **0.1716 ± 0.0875** | **C–O–H** | ***def*(CHO)** | **Glycogen** | **<< 0.05** |
| **880** | **0.5786 ± 0.3124** | **878** | **0.2595 ± 0.1665** | **C–O–P** | **ν(COP)** | **Phosphorylated protein** | **<< 0.05** |

* Mann-Whitney *U* test (two-tailed); significant when *p*<0.05

**Values in bold refer to significantly different peak absorbance between malignant and benign samples (*p*>0.05)

**Abbreviations:** *v*: stretching; δ: bending; γ: wagging, twisting and rocking; *s*: symmetric; *as*: asymmetric; *def*: deformation

***Also, lines 432-443 of page 21 concerning Visual Peak Analysis of spectral data now read:***

“The test of homogeneity demonstrated distinct differences (*p*<0.05) between the absorbance peaks of malignant and benign samples, specifically at bands, 1537 cm^-1^ / 1535 cm^-1^, 1314 cm^-1^ / 1312 cm^-1^, 1051 cm^-1^ / 1030 cm^-1^, and 880 cm^-1^ / 878 cm^-1^. No distinct differences (*p*>0.05) were noted at bands 1636 cm^-1^ /1638 cm^-1^, 1452 cm^-1^ / 1452 cm^-1^, 1401 cm^-1^ / 1397 cm^-1^, 1236 cm^-1^ / 1236 cm^-1^, 1160 cm^-1^ / 1160 cm^-1^. This implies that identified molecular sources that corresponded to the wavenumbers with significant differences were distinct among the two classes. Amide II proteins were found to have significantly decreased absorbances in malignant samples. Phosphorylated protein and glycogen, conversely, were found to have increased absorbance values in malignant samples. This finding suggests that identified molecular sources, amide II, glycogen, and phosphorylated protein, may be considered as a point of comparison between malignant and benign classes.”

*The PCA biplot of malignant and benign samples was also changed due to the modification in the normalization process.* ***Thus, Fig 6 was changed as follow:***

**Fig 6.** **PCA biplot showing the distribution of malignant and benign samples and the variances contributed by each biomolecule.** The red points represent the malignant samples while the blue points represent the benign samples.

***Lines 463-469 of page 23 concerning Principal Component Analysis of spectral data now read:***

“The variation between benign and malignant samples is shown in Fig 6. As presented in the PCA plot, the first principal component F_1_ was associated with 85.65% of the variability, while only 5.67% was associated with the second principal component F_2_. The majority of the benign samples were situated in the negative domain of the F_1_ axis while most of the malignant samples were scattered throughout the F_1_ axis. A clear separation between the two sample classes was not distinct, as shown in the aforementioned figure, hence implying that some malignant and benign samples shared some characteristics and thus difficult to differentiate.”

***Also, lines 716-725 of page 35 concerning the discussion of biomolecular sources of different band positions of malignant spectral data now read:***

“The nine (9) distinct band positions identified by ATR-FTIR spectroscopic analysis elucidated the metabolic changes in malignant samples. Several band positions had shown no significant difference between malignant and benign samples (Table 3). This implies that the spectral data of both classes were difficult to differentiate, especially when using simple linear models. However, there were three (3) band positions that showed significant differences in absorbances.

Band absorbance of 1537 cm^-1^ is within the amide II protein region. There was a significant decrease in amide II proteins in malignant samples compared to benign samples. The decrease in absorbance in the amide band region can be due to proteolysis in the lung cells induced by tumor metabolism [65], resulting in protein remodeling [66,67].”

*Similar to Fig 6, the PCA plot of the discordant malignant and benign samples was changed.* ***Thus, Fig 8 was changed as follow:***

**Fig 8. Distribution of discordant samples from concordant samples via PCA.** The plot shows the diagnosis of the models per discordant samples (dark-colored points) over the distribution of concordant malignant samples (light red) and concordant benign (light blue) samples. The diagnoses of the models for all discordant samples were consistent with the original diagnoses of the study sites.

***Thus, lines 624-631 of page 31 concerning Pseudo-clinical Test now read:***

“Among the specimens, 94 samples (42 malignant; 52 benign) were in diagnostic concordance with the external evaluators and the original diagnosis of the study site; while for the 24 samples, the pathologists had discordant readings. The distribution of discordant samples in the PCA score plot is shown in Fig 8. The majority of the discordant benign and malignant samples were situated similarly within the region of the concordant benign and malignant samples, respectively. Moreover, the discordant readings followed a similar distribution, indicating that they were not outliers. The discrimination of the NN models for all discordant samples was consistent with the diagnoses of the respective study sites.”

Also, for some classifiers, data with entire variables are used, and PCA components are used for others. Although it should not have a big difference in the results, it is better to have similar input to all the classifiers while comparing the performances. Here, the number of variables/features is way more than the number of training samples; hence generalization of the classifier performance is not guaranteed.

**Our answer:**

*We thank the reviewer for the comment above to have a similar input to all classifiers while comparing the performances. As such, we have updated our LDA to include all 462 variables for proper benchmarking. Although the metrics for LDA have been changed, it remains incompetent relative to the NNs and the SVM.*

***Thus, lines 262-272 of page 12 concerning Linear Discriminant Analysis now read:***

“The LDA model was constructed following Fisher’s Criterion [44], $J\left( w \right)=\frac{w^{T}S_{B}w}{w^{T}S_{w}w}$ , where $S_{B}$ and $S_{W}$ denotes the between-class and the within-class covariance matrixes, derived from the training set $X_{TR}\in X_{SD}$, respectively; where $X_{TR}$ is a matrix of samples having 462 variables. Here, $w$ is the eigenvector with the highest eigenvalue derived from the solution of $\frac{dJ(w)}{dw}$ which is equal to $eig(S_{C}^{-1}S_{B})$. The probability of malignancy, $p(X)$, for each sample $X^{(i)}$ was derived using the formula

$p\left( X \right)=softmax\left( {[f}_{norm}\left( s,\mu_{B},\sigma\right), f_{norm}\left( s,\mu_{M},\sigma\right)] \right) | s=X\times w$	(4)

where the function $f_{norm}$ denotes the value of a Gaussian probability density function at a point $s$, with means $\mu_{B}$ and $\mu_{M}$ which are the benign and the malignant mean projected values, respectively, from the training set, and $\sigma$ is the linear distance between $\mu_{B}$ and $\mu_{M}$. The process was repeated for 50 trials to ensure stability.”

***Also, Table 7 was changed as follow:***

**Table 7. Mean and standard deviation of diagnostic performance of all the machine learning models.**

|  | **FNN 2** | **FNN 4** | **FNN 8** | **RNN** | **CNN** | **DT** | **RF** | **NB** | **LDA** | **LR** | **SVM** |
| --- | --- | --- | --- | --- | --- | --- | --- | --- | --- | --- | --- |
| *AUC (%)* | 92.41 ± 10.28 | 92.85 ± 9.98 | 92.77 ± 9.62 | 90.40 ± 11.62 | 92.28 ± 7.36 | 78.93 ± 19.87 | 92.15 ± 13.79 | 77.91 ± 21.22 | 62.92 ± 13.44 | 82.16 ± 19.84 | 99.38 ± 1.97 |
| *ACC (%)* | 98.41 ± 4.07 | 98.39 ± 3.57 | 97.61 ± 4.91 | 95.98 ± 6.25 | 98.45 ± 1.72 | 77.58 ± 16.91 | 85.87 ± 15.11 | 75.13 ± 19.14 | 65.45 ± 14.60 | 72.19 ± 18.68 | 94.38 ± 9.69 |
| *PPV (%)* | 94.92 ± 9.49 | 96.03 ± 8.63 | 96.48 ± 8.01 | 90.91 ± 11.99 | 96.62 ± 2.30 | 73.95 ± 30.28 | 83.55 ± 26.14 | 64.65 ± 33.42 | 59.54± 20.84 | 65.25 ± 36.13 | 93.85 ± 14.92 |
| *NPV (%)* | 92.79 ± 11.95 | 93.57 ± 11.28 | 93.22 ± 10.75 | 94.03 ± 9.26 | 90.50 ± 11.92 | 80.11 ± 21.98 | 87.60 ± 18.36 | 82.21 ± 22.01 | 70.25 ± 15.13 | 94.89 ± 30.57 | 94.57 ± 12.11 |
| *SR (%)* | 94.60 ± 11.41 | 94.67 ± 11.57 | 95.30 ± 10.64 | 86.63 ± 17.58 | 96.01 ± 3.09 | 84.36 ± 18.04 | 90.78 ± 14.34 | 79.79 ± 18.68 | 69.08 ± 14.50 | 74.97 ± 25.98 | 96.75 ± 8.03 |
| *RR (%)* | 89.84 ± 16.36 | 90.88 ± 16.13 | 90.10 ± 15.66 | 92.87 ± 13.18 | 89.21 ± 12.93 | 73.35 ± 28.25 | 83.74 ± 24.06 | 71.10 ± 33.83 | 62.13± 17.14 | 64.49 ± 35.74 | 94.46 ± 12.64 |

$-$You can either remove equations for classic classifiers or write them correctly. Text at line 269 is not very clear; it does not properly connect with the inline equation 7. Also, in line 277, the authors talk about equations but instead provide references. Again, the authors discuss terms from these equations which are not present in the manuscript. Again at line 280, α is explained without any mention of α before that.

**Our answer:**

*We thank the reviewer for the valuable comment. We have removed the discussions about the classical machine learning models and replaced them with citations on the software packages that were used in the study. Also, we have updated our algorithm in LDA to include all 462 variables for proper benchmarking. The changes were made on lines 261-316 of the manuscript*

***Thus, lines 262-272 of page 12 concerning Classic Classifiers now read:***

**“Linear discriminant analysis**

The LDA model was constructed following Fisher’s Criterion [44], $J\left( w \right)=\frac{w^{T}S_{B}w}{w^{T}S_{w}w}$ , where $S_{B}$ and $S_{W}$ denotes the between-class and the within-class covariance matrixes, derived from the training set $X_{TR}\in X_{SD}$, respectively; where $X_{TR}$ is a matrix of samples having 462 variables. Here, $w$ is the eigenvector with the highest eigenvalue derived from the solution of $\frac{dJ(w)}{dw}$ which is equal to $eig(S_{C}^{-1}S_{B})$. The probability of malignancy, $p(X)$, for each sample $X^{(i)}$ was derived using the formula

$p\left( X \right)=softmax\left( {[f}_{norm}\left( s,\mu_{B},\sigma\right), f_{norm}\left( s,\mu_{M},\sigma\right)] \right) | s=X\times w$	(4)

where the function $f_{norm}$ denotes the value of a Gaussian probability density function at a point $s$, with means $\mu_{B}$ and $\mu_{M}$ which are the benign and the malignant mean projected values, respectively, from the training set, and $\sigma$ is the linear distance between $\mu_{B}$ and $\mu_{M}$. The process was repeated for 50 trials to ensure stability.

**Support vector machine**

The designed support vector machine (SVM) is a linear SVM. The SVM is designed using the elements of the training set $X_{TR}\in X_{SD}$, by considering an unconstrained Lagrange optimization problem [45,46]. To determine sub-optimal values for the parameters of the SVM, stochastic gradient descent (SGD) was used. A grid search was performed to select the best learning rate for the SGD; the validation set accuracy was considered as the optimization metric which determined the superiority of one model over the other. The output probability diagnosis of the model for benign and malignant cases was computed using Platt’s method [47]. The process was repeated for 50 trials to ensure stability.

**Logistic regression**

The designed logistic regression (LR) model is a $462$-input classifier with an output probability quantifying the likelihood of a sample to be malignant. In training the model, SGD was used over a training set, $X_{TR}\in X_{SD}$ using the binary cross-entropy function as the loss function. A grid search was performed, similar to that of the SVM, to optimize the SGD learning rate. The process was repeated for 50 trials to ensure stability.

**Decision tree and random forest**

The classification and regression trees (CART) algorithm were used to generate decision trees (DTs) of binary splits. The Gini’s diversity index [48] was used to find the best input variable ($\omega=X^{T} | \omega\in\mathbb{R}^{462\times N}$) for splitting the training set for each iteration of branching. Since the values of $\omega$ are continuous, the best value of separation was identified by considering the variable $\omega_{j}\in\omega$ having the least $Gini$ metric [49]. The branching was recursively performed for each newly created node until the performance in the validation set accuracy decreased. The process was repeated for 50 trials to ensure stability.

The designed random forest (RF) model utilized the creation of trees following the previously discussed. The diagnosis of the RF was determined as the prevailing diagnosis made by its constituent bags of DTs. To determine the optimum number of trees $N_{RF}$for the RF, a grid search from 3 to 100 trees was performed. The validation set accuracy was considered as the optimization criteria of the search. Each simulation was repeated over 50 times for each iteration to ensure stability. The average performance metric over the 50 trials served as the final performance metric of the RF with the number of trees set to $N_{RF}$.

**Naïve bayes**

The designed NB is a classifier of 2 classes. For each input variable $\omega_{j}\in\omega$, the best value of separating the two sub-classes was determined. The algorithm for finding the best value of separation is the same as that of the DT and RF designs where the Gini’s index was used. The NB classifier outputs a malignant diagnosis when the probability obtained was more than 50%, otherwise the diagnosis is benign. In order to determine the optimal number of input for the classifier, the number of inputs was increased from 3 to $462$, where the inputs of the least $GINI{(j)}_{t}$ value were considered first. The optimization was terminated at the iteration where the validation accuracy of the model started to decrease. Each iteration of was repeated for 50 trials, where the average validation accuracy from the 50 trials was the considered optimization metric criterion. The final NB constituted to the design with the highest average validation accuracy.”

$-$ The manuscript makes it hard to understand what you mean by individuals in GA; please describe it explicitly.

**Our answer:**

*We thank the reviewer for the valuable comment to explain in detail what an individual pertains to. Hence, we have edited the manuscript following the reviewer’s recommendations. The revised* ***‘Genetic algorithm design’ section, in lines 348-352 of pages 15-16*** *now reads:*

An individual is a solution that is made up of a unique combination of NN parameters. The generation of individuals begins once each parameter has been accurately defined. Individuals are created at random in this method. This stochastic generation of individuals refers to the selection of a random value for each parameter of each individual.
